# Factors regulating microglia activation

**DOI:** 10.3389/fncel.2013.00044

**Published:** 2013-04-23

**Authors:** Katrin Kierdorf, Marco Prinz

**Affiliations:** ^1^Institute of Neuropathology, University of FreiburgFreiburg, Germany; ^2^Faculty of Biology, University of FreiburgFreiburg, Germany; ^3^BIOSS Centre for Biological Signalling Studies, University of FreiburgFreiburg, Germany

**Keywords:** microglia, activation, development, transcription factors, silencing

## Abstract

Microglia are resident macrophages of the central nervous system (CNS) that display high functional similarities to other tissue macrophages. However, it is especially important to create and maintain an intact tissue homeostasis to support the neuronal cells, which are very sensitive even to minor changes in their environment. The transition from the “resting” but surveying microglial phenotype to an activated stage is tightly regulated by several intrinsic (e.g., Runx-1, Irf8, and Pu.1) and extrinsic factors (e.g., CD200, CX_3_CR1, and TREM2). Under physiological conditions, minor changes of those factors are sufficient to cause fatal dysregulation of microglial cell homeostasis and result in severe CNS pathologies. In this review, we discuss recent achievements that gave new insights into mechanisms that ensure microglia quiescence.

## MICROGLIA – GATEKEEPER OF TISSUE HOMEOSTASIS IN THE CNS

Tissue macrophages are found in virtually all organs of the body. In the central nervous system (CNS), specialized tissue macrophages were first identified within the “third element,” or “mesoglia,” by Pio del Rio-Hortega (1882–1945). He first characterized a small cell in the neuroectodermal tissue, which apparently was of mesodermal origin and seemed to be related to other tissue macrophages in the body (del Rio-Hortega, 1919, 1932). The specialized tissue macrophage of the CNS is known today as microglia.

The postulated mesodermal origin of microglia by Pio del Rio-Hortega was under investigation for several decades. However, the issue about the exact origin of microglia was not solved. An increasing number of studies pointed to a very early colonization of the CNS by mesodermal progenitors (Kaur et al., 2001) and indicated that microglial progenitors arise from the yolk sac (Alliot et al., 1999). More recently, we and others showed that microglia are derived from the primitive hematopoiesis in the yolk sac and excluded a contribution of definitive hematopoietic stem cells (HSCs) to the generation of microglia (Ginhoux et al., 2010; Schulz et al., 2012). It was further shown that microglia are derived from an uncommitted F4/80-negative erythromyeloid precursor in the yolk sac that develops via immature F4/80^-^CX_3_CR1^-^ myeloid progenitor subsets into F4/80^+^CX_3_CR1^+^ mature macrophages, which finally colonize the CNS to give rise to microglia (Kierdorf et al., 2013).

Upon infection or insults within the adult CNS parenchyma, microglia are rapidly activated and efficiently phagocytose pathogens and dying cells (Hanisch and Kettenmann, 2007; Ransohoff and Perry, 2009). Furthermore, microglia release many effector molecules for the recruitment of other immune cells from the blood to limit infections in the CNS, or which serve as antigen presenting cells of phagocytosed material (Saijo and Glass, 2011). In addition, they help in the regeneration of damaged tissue by secretion of growth factors and anti-inflammatory molecules (Saijo and Glass, 2011). Therefore, microglia are indispensable in the adult CNS as stabilizers and modulators of tissue homeostasis under physiological conditions.

Microglia are well integrated in the neuronal glial network of the healthy CNS. They are distributed in all brain regions with varying density between 5% in the *corpus callosum* and 12% in the *substantia nigra* (Lawson et al., 1990). However, the reason for this difference in cell frequency has not been resolved. The morphology of a “resting” microglial cell is characterized by a very small cell soma with elongated ramified processes (Cuadros and Navascués, 1998). Under healthy conditions, microglial cell processes do not overlap with processes of neighboring cells and each cell seems to have a scavenger function for its own immediate area. The position of the cell soma remains stable, whereas the processes of the resting microglia are continuously elongating and retracting to explore the tissue environment. *In vivo* imaging of microglia in intact brain tissue demonstrated highly dynamic processes which continuously scan the surrounding microenvironment (Nimmerjahn et al., 2005). Upon recognition of a pathogen or other inflammatory stimuli, microglia can rapidly retract their processes and become efficient mobile effector cells (Davalos et al., 2005). These observations highlighted the important immune surveillant function of microglia in the healthy CNS parenchyma. In general, microglia activation is triggered by a plethora of well described subsets of immune receptors such as Toll-like receptors (TLRs), scavenger receptors, and numerous cytokine and chemokine receptors.

In this review, however, we focus on several distinct exogenous as well as endogenous signals and factors that are important for the maintenance of the “resting” state of microglia. First, we describe the interaction between microglia and their surrounding cells, such as neurons, which have a major role in transmitting survival and inhibitory signals to resting microglia. Next, we discuss endogenous microglial signals, mainly transcription factors, which modulate the transcriptional program in these cells that either lead to changes in microglial features or maintain their resting state. Small perturbations in one of these signaling cascades can lead to a spontaneous activation of microglia without any infection or injury to the CNS. However, this spontaneous activated phenotype may be harmful to the neuronal network via induced major changes in the tissue environment, and result in severe damage of the neuronal integrity and function.

## EXOGENOUS SIGNALS FOR MICROGLIA ACTIVATION

In the non-diseased adult CNS, microglia communicate with the surrounding glial cells as well as with neighboring neurons. This communication is enabled by a versatile subset of different cell surface molecules on the microglial cell membrane. Indeed, most of the surface molecules of microglia belong to the families of cytokine receptors, scavenger receptors, and pattern recognition receptors (PRRs), as well as to the chemokine receptors, which recognize pro-inflammatory mediators upon inflammation or infection. Most of these receptors are binding ligands that are secreted by, or expressed on the membrane of healthy neurons. Activation of these receptor subsets by inflammatory molecules or pathogens result in a rapid activation of the “resting” microglia to a motile effector cell which contributes to the ongoing inflammation. However, for a small heterogeneous subset of molecules known as “inhibitory molecules” on the surface of microglia, their ligand recognition and binding does not result in a pro-inflammatory activation (Ransohoff and Perry, 2009). Otherwise, the binding of these ligands is necessary for keeping up the resting ramified phenotype of microglia in the healthy CNS (Linnartz and Neumann, 2012).

**FIGURE 1:**
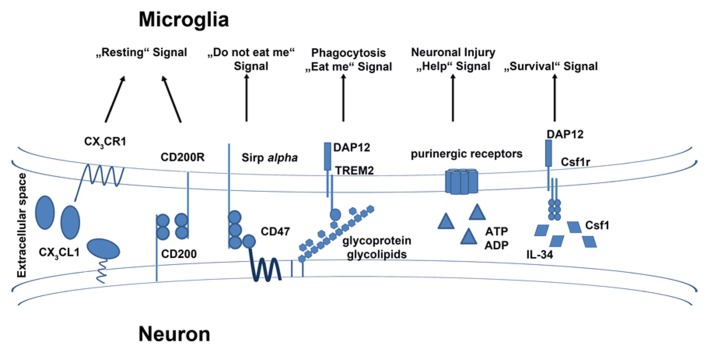
**Exogenous signals and their receptors on microglia.** Microglia are equipped with a group of surface receptors which trigger signals in microglia under “resting” conditions. Many of these signals are ligands which are released or expressed on the surface of neurons. Inhibitory receptors like CX_3_CR1, CD200R, and CD172a/Sirp *alpha* have their ligands on the surface of healthy neurons. A loss of the signal also indicates a loss of neuronal integrity. Receptors such as TREM2 and purinergic receptors are essential to mimic neuronal injury and induce phagocytotic and anti-inflammatory functions in microglia. The crosstalk between microglia and neurons is also important for the survival of microglia. Neurons release factors like IL-34 and Csf1 which bind to Csf1r on the surface of microglia and induce cell survival or proliferation.

The surface molecule CD200 is widely expressed not only on neurons, but also on astrocytes and oligodendrocytes (Barclay et al., 2002). Its receptor CD200R is exclusively expressed on macrophages in the CNS, including microglia. The interaction of neuronal CD200 with CD200R leads to inactivation of microglia and keeps them in a resting state (Hoek et al., 2000; Biber et al., 2007) (**Figure 1**). Analysis of microglia of CD200-deficient mice revealed a less ramified morphology with shorter processes and upregulation of CD45 (leukocyte common antigen) and CD11b (complement receptor 3/integrin α_m_β_2_), which are also markers of activation (Hoek et al., 2000). Additionally, microglia in non-immunized CD200-deficient animals seemed to form aggregate-like structures, which are typically only found in neurodegenerative disease with strong microglial activation. Following facial nerve axotomy, a model for local neuronal degeneration, CD200-deficient neurons elicited an accelerated microglial response in the lesioned nucleus. In the animal model for multiple sclerosis (MS), experimental autoimmune encephalomyelitis (EAE), deficiency of CD200 resulted in a more rapid onset of disease (Hoek et al., 2000). These findings indicate that without the CD200–CD200R signaling, microglia develop an activated phenotype in the CNS.

The G-protein-coupled seven-transmembrane chemokine receptor, CX_3_CR1, is expressed on monocytes, macrophages, dendritic cells (DCs), as well as on natural killer (NK) cells. In the CNS parenchyma, CX_3_CR1 expression is restricted to the macrophage population, e.g., microglia (Jung et al., 2000). Its only known ligand, CX_3_CL1, is expressed on different neuronal subsets in the adult CNS (Kim et al., 2011). Also called fractalkine or neurotactin, CX_3_CL1 was first described as a member of a new chemokine class with a specific cysteine motif containing three amino acids separating both cysteines in the chemokine domain (Bazan et al., 1997; Pan et al., 1997). The ligand can be found in a secreted form as well as in a membrane-bound variant (Hughes et al., 2002). Harrison et al. (1998) showed that the binding between of neuronal CX_3_CL1 to the microglial CX_3_CR1 seems to play a fundamental role in the interaction of neurons and microglia in the healthy and diseased brain. Furthermore, they postulated in this study that CX_3_CL1 binding to CX_3_CR1 is a fundamental signaling pair in neurophysiology.

Cardona et al. (2006) also found high levels of secreted CX_3_CL1 in the CNS parenchyma under healthy conditions. They further demonstrated that CX_3_CR1-deficient microglia show an over-activated phenotype in three different diseases models. The loss of fractalkine signaling led to an enhanced neuronal cell death in animal models for Parkinson’s disease and other motor neuron disorders. The accelerated neurotoxicity of the CX_3_CR1-deficient microglia seemed to worsen neurodegenerative diseases. Nevertheless, several studies on the role of CX_3_CR1-deficiency in animal models for Alzheimer’s disease (AD) revealed quite divergent results (Fuhrmann et al., 2010; Lee et al., 2010; Liu et al., 2010; Prinz et al., 2011). In AD, CX_3_CR1 deficiency appeared to be beneficial by resulting in reduced neuronal loss and improved behavioral deficits. Altogether, these results indicated that CX_3_CR1-deficiency has several effects on the course and pathology of inflammatory or neurodegenerative CNS diseases. Therefore, neuron–microglia communication via CX_3_CL1 and CX_3_CR1 could play different roles under inflammatory and neurodegenerative CNS conditions (Prinz and Priller, 2010).

Notably, recent investigations showed a pivotal role for the CX_3_CR1–CX_3_CL1 signaling under physiological conditions (**Figure 1**). In the postnatal brain, the formation of mature neural circuits depends on the elimination of redundant synapses. Little was known about the mechanisms of this process called “synaptic pruning” (Hua and Smith, 2004). Recent investigations showed that the ligand CX_3_CL1 is highly expressed during this time of synapse maturation (Paolicelli et al., 2011). The authors could elegantly demonstrate that microglia are in direct contact with these synapses and remove unwanted synapses by phagocytosis. This study highlighted the non-redundant role of CX_3_CR1 on microglia for this process. CX_3_CR1-deficient animals showed a reduced number of microglia during the first weeks after birth (Paolicelli et al., 2011). However, during the embryonic phase and in adult animals there was no alteration in microglial cell numbers detected in these animals (Kierdorf et al., 2013). The authors pointed out that CX_3_CR1 is an important regulator of microglial surveillance (Paolicelli et al., 2011). Reduced microglial cell number in CX_3_CR1-deficient animals resulted in the development of immature neuronal circuits. In contrast, CX_3_CR1-deficient animals revealed a high density of spines and functional excitatory synapses, which are more known to be related to a mature phenotype than delayed brain development.

In addition, another study reported that CX_3_CR1-deficient animals exhibited an inhibition in microglial recruitment to forming synapses in the barrel field of the somatosensory cortex, leading to an abnormal synapse formation in this area (Hoshiko et al., 2012). Therefore, interaction of neurons and microglia appears to be essential for proper synapse formation in the postnatal cortex.

Earlier publications did not indicate an essential role of CX_3_CR1 in the healthy brain. Most studies were dealing with the role of this receptor under inflammatory conditions (Jung et al., 2000; Cardona et al., 2006). Recent investigations in healthy CX_3_CR1-deficient animals showed a harmful effect of this mutation for adult neurogenesis and hippocampal circuit integrity. The number of neuronal precursors in the hippocampus was massively decreased and led to diminished adult neurogenesis (Bachstetter et al., 2011). Reintroducing CX_3_CL1 in the hippocampus of aged animals could rescue of the decreasing neurogenesis generally observed in aging animals. Furthermore, a reduction of CX_3_CR1 in adult animals resulted in a decrease in neurogenesis. The authors showed that a lower level of neurogenesis is mediated by highly elevated levels of hippocampal pro-inflammatory cytokines such as interleukin (IL)-1β, which is secreted by microglia, and is toxic to the developing neuronal progenitors. This supports the role of CX_3_CR1 signaling in keeping microglia in a quiescent state.

Another study further evaluated this theory and found that CX_3_CR1-deficient animals display cognitive impairment (Rogers et al., 2011). They established performance deficits in fear conditioning as well as in the Morris water maze, which is associated with learning, in CX_3_CR1-deficient mice. Mice lacking this receptor had significant impairment in long-term potentiation, which is widely considered to underlie learning and memory. The authors demonstrated an essential role of IL-1β in these CX_3_CR1-mediated impairments by reversing the deficits of CX_3_CR1-deficiency via application of IL-1R antagonists.

There are many other inhibitory surface receptors or molecules on the surface of microglia which are mostly interacting with ligands secreted or expressed on the surface of neurons, such as CD47. CD47 is expressed ubiquitously, including on neurons, transmits a “do not eat me” signal to the microglia via CD172a/Sirp *alpha* (van Beek et al., 2005; Biber et al., 2007) (**Figure 1**). Another glycoprotein identified on microglia is known as the triggering receptor expressed on myeloid cells 2 (TREM2), which was linked to an anti-inflammatory phenotype (Colonna, 2003). TREM2 is associated with the adaptor protein DNAX-activating protein of 12-kDa (DAP12), which transmits the signal from the receptor to the intracellular signaling cascade. TREM2 is essential for phagocytosis of, for example, apoptotic cell membranes by microglia (Neumann and Takahashi, 2007). Furthermore, TREM2 was shown to have beneficial effects in autoimmune CNS demyelination (Takahashi et al., 2007). Here, myeloid precursors from bone marrow cultures, which were lentivirally transduced to overexpress TREM2, were transplanted into EAE animals. The resultant disease amelioration in this animal model for MS indicated that TREM2 may be the key receptor that triggers tissue repair and limits CNS damage. Mutations in TREM2 or DAP12 are associated with a severe neurodegenerative disease known as polycystic lipomembranous osteodysplasia with sclerosing leukoencephalopathy (PLOSL), which is further characterized by a form of early onset dementia and formation of bone cysts (Paloneva et al., 2001; Klünemann et al., 2005).

Microglia also express receptors that trigger essential cellular survival and developmental signals. One of the receptors which plays a major role in microglial development and survival is the receptor for colony stimulating factor 1 (Csfr1), which is also known to regulate the differentiation and survival of peripheral macrophages (Chitu and Stanley, 2006) (**Figure 1**). Csf1r binds Csf1, which is important for the maintenance of many macrophage subsets (Cecchini et al., 1994). Csf1r-deficient animals show deficiency in several subsets of mononuclear phagocytes and microglia are completely absent in these mice (Dai et al., 2002; Ginhoux et al., 2010). Ginhoux et al. (2010) further showed that the development of microglia and primitive yolk sac macrophages is completely dependent on Csf1r signaling. However, the Csf1^op/op^ mouse strain with a natural occurring null mutation in Csf1 did not reveal the same severe phenotype observed in the Csf1r-deficient animals (Yoshida et al., 1990). In fact, detailed examination of Csf1^op/op^ mice revealed the presence of microglia but at reduced numbers (Blevins and Fedoroff, 1995). These findings clearly indicated that survival and maintenance of microglia are majorly influenced by Csf1r function. However, Csf1r may have Csf1-independent functions in microglial homeostasis, suggesting the existence of another ligand for this receptor. Indeed, (Lin et al., 2008) discovered another ligand of the Csf1r known as IL-34. It was shown that IL-34 and Csf1 share some similar signaling functions via Csf1r and that both ligands can compensate for each other (Wei et al., 2010). However, both ligands showed a differential expression pattern *in vivo*. Wang et al. (2012) generated IL-34-deficient animals with an insertion of the LacZ reporter gene in the IL-34 gene locus, allowing its expression to be traced to neurons. They showed that mostly microglia and Langerhans cells of the epidermis are affected by the loss of IL-34. IL-34-deficient animals had reduced numbers of microglia and were consequently more susceptible to viral infections in the CNS. Similar results were recently obtained by Greter et al. (2012). They demonstrated that IL-34 is expressed by specific neuronal subsets restricted to defined brain regions such as the cortex and hippocampus (Greter et al., 2012). The authors found that microglial development was not influenced by IL-34 deficiency. In contrast, microglial cell number was decreased in specific regions of the adult brain. These results indicated that IL-34 plays a role in the adult brain for microglial survival and homeostasis. In another recent study, Erblich et al (2011) analyzed microglia in Csf1^op/op^ mice and Csf1r-deficient animals. They observed a reduced number of microglial cell in the absence of Csf1, and a complete loss of microglia in Csf1r-deficient animals. These severe defects in microglial developments resulted in disturbed brain development with a prominent phenotype of a thinned cortex. Additionally, a recent study showed that the signaling of both IL-34 and Csf1 are important for microglial proliferation upon neurodegeneration during prion infection and AD (Gómez-Nicola et al., 2013). Injection of a blocking antibody against Csf1r on activated microglia induced a strong reduction of proliferating cells in both diseases. In turn, microglial proliferation could be induced by administration of Csf1 and IL-34, whereas IL-34 showed a stronger proliferative capacity then Csf1. In addition to the studies from Wang et al. (2012) and Greter et al. (2012), it was shown here that not only are neurons a source of IL-34, astrocytes could also express this ligand under neurodegenerative conditions. Therefore, the sources of IL-34 might differ depending on the cellular conditions and the time points of investigation. Interestingly, heterozygous mutations in the Csf1r locus can be found in patients with hereditary diffuse leukoencephalopathy with spheroids, characterized by demyelination of the cerebral white matter and formation of spheroids which lead to progressive cognitive and motor dysfunction (Rademakers et al., 2012). However, it is still unclear, what roles IL-34 and Csf1 play in microglial development or homeostasis in these patients. Future studies will elucidate the detailed function of these important physiological signaling molecules in microglia.

Release of nucleotides such as adenosine triphosphate (ATP) in the CNS mimics an inflammatory insult in the parenchyma, especially after nerve injury, and could efficiently activate microglia (Davalos et al., 2005). This activation is mediated by several purinergic receptors on the microglial surface. Microglia are equipped with a wide range of ionotropic (P2X_4_ and P2X_7_) and metabotropic (P2Y_1_, P2Y_2_, and P2Y_12_) purinergic receptors (Inoue, 2002; Haynes et al., 2006). Many studies focused on the role of these nucleotides for microglial activation and showed an important function in their early response to injury (Haynes et al., 2006; Ohsawa et al., 2007; Ulmann et al., 2008). Nucleotides are versatile effector molecules which initiate the fast recruitment of microglia to injury sites for the release of neurotrophic factors (Haynes et al., 2006; Ohsawa et al., 2007; Ulmann et al., 2008). The purinergic receptors seem to be another subset of microglial surface receptors which are important for the maintenance of injured neurons and facilitating tissue homeostasis by microglia (Inoue, 2002).

Therefore, it can be assumed that microglia are very important for the development and maintenance of the neuronal network. As soon as there is a microglial activation without any insult or infection that is solved, this activation can be disruptive and harmful for developing and already existing neuronal networks.

## ENDOGENOUS FACTORS REGULATING MICROGLIA ACTIVATION

The state of the microglial cell is not only regulated by exogenous signal via surface receptors. Their activation and maturation states are tightly controlled by a subset of endogenous transcription factors. Factors like Runt-related transcription factor 1 (Runx1), ETS (E-twenty six) family transcription factor Pu.1, and interferon regulatory factor 8 (Irf8) are indispensable regulators of the differentiation process during embryonic development (Ginhoux et al., 2010; Kierdorf et al., 2013).

Runx1 was already known for its crucial function during definitive hematopoiesis. Loss of Runx1 leads to a complete lack of HSC development from the aortic endothelium, accompanied by an abnormal fetal liver hematopoiesis (Okuda et al., 1996; North et al., 1999). Okuda et al. (1996) generated Runx1-deficient animals and showed that they die very early during embryonic development at around 12.5 days post conception (dpc). Another study indicated that Runx1-deficient embryos also suffer from necrosis and hemorrhages in the CNS (Wang et al., 1996). Cell tracing studies in the early mouse embryo revealed that Runx1 is a transcription factor which is already expressed in hematopoietic progenitors in the extraembryonic blood islands (Samokhvalov et al., 2007). Ginhoux et al. (2010) used an inducible Cre-recombinase under the control of the Runx1 promoter for their pulse labeling experiments during early embryonic stages. Here, they targeted microglia by pulse labeling Runx1^+^ progenitors in the yolk sac around 7.5 dpc, since Runx1 is expressed in early microglial progenitors. The decisive role of Runx1 in myelopoiesis was recently described in zebrafish (Jin et al., 2012). Both transcription factors Pu.1 and Runx1 were shown to act in a negative feedback loop for the regulation of Pu.1 expression levels and thereafter regulating myeloid cell fate (Jin et al., 2012). These findings indicated a key role of Runx1 for myeloid and microglia development. However, it was unclear at this point whether Runx1 is also involved in the cell homeostasis of microglia. In a transcriptome analysis of laser-microdissected microglia from the corpus callosum of rats, Runx1 was found to be down regulated in ramified microglia of 4 week old rats, compared to amoeboid microglia of 5 days old pubs (Parakalan et al., 2012). Zusso et al. (2012) performed a detailed analysis for the function of Runx1 in postnatal microglia. They showed that Runx1 is not only a regulator of differentiation, but further regulates proliferation and homeostasis of postnatal microglia. They further suggested that Runx1 might play an important function in microglia by modulating the transition of amoeboid microglia to ramified ones. Therefore, Runx1 is a non-redundant transcription factor that is important for the activation and resting states of microglia(**Table 1**).

**Table 1:** Transcription factors regulating microglia development and homeostasis.

Transcription factor	Microglia development	Microglia homeostasis
c-myb	→ Independent of c-myb (Schulz et al., 2012; Kierdorf et al., 2013)	→ After induced ablation of c-myb, no change in microglia cell number (Schulz et al., 2012)
Runx-1	→ Expressed on microglia progenitors in the yolk sac (Ginhoux et al., 2010)	→ Regulating proliferation and tissue homeostasis of postnatal microglia (Zusso et al., 2012)
		→ Transition of amoeboid to ramified morphology (Zusso et al., 2012)
Pu.1	→ Absence of microglia progenitors in the yolk sac; no development of microglia (Scott et al., 1994; McKercher et al., 1996; Beers et al., 2006; Schulz et al., 2012; Kierdorf et al., 2013)	→ No direct evidence yet for a functional role during microglia cell homeostasis
Irf8	→ Reduced number of microglia and microglial progenitors in the yolk sac (Kierdorf et al., 2013)	→ Dysregulation of microglial cell morphology and function (Horiuchi et al., 2012; Minten et al., 2012)
		→ Defects in microglial activation (Masuda et al., 2012)
Hoxb8	→ No direct evidence yet for a functional role during microglia development	→ Mutant Hoxb8 microglia induce pathological grooming in mice (Chen et al., 2010)

The transcription factor Pu.1 is a master regulator of myeloid development which is already required during the first stages of myeloid differentiation programs. Pu.1-deficient animals indicated a fundamental role of Pu.1 for myeloid and microglial development (Scott et al., 1994; McKercher et al., 1996). These animals died during the first days after birth due to severe septicemia. Their lifespan could be extended by antibiotic treatment or bone marrow transplantation (Beers et al., 2006). Mature B cells and myeloid cells are completely missing in these mice. Examination of several tissues revealed a complete loss of tissue macrophages such as Kupffer cells, microglia, or other tissue macrophages (Schulz et al., 2012). Another study demonstrated that CNS cultures from Pu.1-deficient animals showed reduced proliferation of cortical precursors and reduced astrogliogenesis (Antony et al., 2011). Until now, there is no direct evidence whether Pu.1 is also involved in the normal homeostasis of adult microglia. However, several studies suggest a regulatory function in the microglia/macrophage activation state. Ponomarev et al. (2011) down regulated Pu.1 in macrophages by overexpression of miRNA-124. The reduced levels of Pu.1 led to deactivated phenotype of macrophages. Further studies are needed to verify the function of Pu.1 in activation and homeostasis of microglia (**Table 1**).

Recent investigations on the development of microglia showed that microglial development is tightly regulated by distinct transcriptional programs. Schulz and colleagues defined microglia as myeloid cells that develop from a HSC-independent progenitor, independent of the transcription factor c-myb, which, however, is indispensable for the development of definitive HSCs (Mucenski et al., 1991; Schulz et al., 2012). Microglia developed normally in c-myb-deficient animals, indicating that c-myb is not regulating microglial development (**Table 1**). Furthermore, they showed that an induced ablation of c-myb in adult mice did not affect adult microglia. C-myb could therefore be considered a transcription factor that is redundant for microglia development and homeostasis.

In a very recent study on microglial development, a detailed characterization of microglial progenitors and factors which are important for their development was performed (Kierdorf et al., 2013). The transcriptional programing of microglial development is tightly regulated by Pu.1 and the myeloid transcription factor Irf8. Irf8 is a transcription factor which is known to be important for the development of B cells and myeloid cells in the bone marrow (Holtschke et al., 1996). It was shown that Irf8-deficient animals have a severe defect in the generation of mature myeloid cells, finally resulting in chronic myelogenous leukemia (CML)-like symptoms (Holtschke et al., 1996). Furthermore, Irf8-deficient animals were more susceptible to infections (Holtschke et al., 1996). Mutations in the Irf8 locus have been found to result in severe immunodeficiency in humans (Hambleton et al., 2011). These patients had decreased mature DCs and monocytes in the blood and suffered from recurrent bacterial infections after Bacillus Calmette–Guérin (BCG) vaccination. The authors described two mutations in the DNA-binding domain of Irf8 which were shown to induce a reduced binding to target gene promoter regions like IL-12 and inducible nitric oxide synthetase (iNOS; Hambleton et al., 2011). Additionally, Irf8 was found to be a susceptibility gene for autoimmune diseases such as Lupus erythematodes and MS (De Jager et al., 2009; The International Multiple Sclerosis Genetics Consortium, 2011; Lessard et al., 2012).

Besides the detrimental role of Irf8 for microglial and myeloid development, recent studies indicated a role of Irf8 in adult microglia homeostasis and activation (**Table 1**). One report demonstrated the involvement of Irf8 for the activation of microglia during nerve injury (Masuda et al., 2012). A massive upregulation of this transcription factor upon lesion induction was detected. Irf8-deficient animals showed less hypersensitivity after nerve injury, decreased activation marker levels in microglia, and no changes in microglial proliferation. In this study, no morphological abnormalities of Irf8-deficient microglia were described. In contrast, two recent studies showed that loss of Irf8 has severe effects on microglia already under physiological conditions. Horiuchi et al. (2012) reported no changes in cell number but found an abnormal cell morphology with reduced ramification and altered expression of ionized calcium-binding adapter molecule (Iba)-1. The authors further described reduced microglia proliferation in culture and diminished phagocytotic capacity in the absence of Irf8. In addition, the authors investigated microglial activation and detected an altered cytokine expression level similar to Masuda et al. (2012). Another study found increased microglial cell numbers and showed gross alterations in morphology and surface area in the absence of Irf8 (Minten et al., 2012).

Another important transcription factor essential for microglial homeostasis is Hoxb8. Hoxb8-deficient animals showed excessive grooming behavior (Greer and Capecchi, 2002). Chen et al. (2010) were able to define Hoxb8 expression in the adult CNS in regions associated with the grooming behavior in mice. In humans, these brain areas correspond to the obsessive-compulsive disorder (OCD) circuitry. OCD is a condition that is often characterized by excessive behaviors dealing with cleanliness, including grooming. Whereas the exact microglia-mediated mechanisms of this pathological grooming were not identified in this study, the authors could elucidate a role of Hoxb8 mutant microglia in a behavioral disorder. Hoxb8 was found to be expressed only in microglia in the CNS, but just in approximately 40% of the cell population which should be derived from bone marrow cells as the authors stated in this study. Loss of Hoxb8 led to a slightly reduced microglial cell number, but no obvious change in cell morphology. The authors showed that wild type bone marrow transplantation in Hoxb8 mutants after total body irradiation rescued the pathological grooming behavior. The authors obtained surprisingly high engraftment rates (up to 30%) of infiltrating bone marrow-derived phagocytes (BMDPs) in the mutant CNS. Furthermore, when they restricted the Hoxb8 deletion to the hematopoietic system, they found the same pathological grooming behavior and hair loss symptoms. This study is one of the first that shows that mutations and defects in microglia can result in prominent behavioral syndromes in mice. Deciphering novel functions of microglia for psychiatric disorders and behavioral anomalies will open new avenues in neuroimmunology in the future.

We conclude that microglia have different surface receptors that are essential for microglial cell homeostasis by regulating either survival and or activation properties. Dysregulation of these receptors induce severe changes in the microglia, which could potentially be harmful for neuronal networks, and result in developmental defects and/or neuropathological changes in the adult CNS.

## Conflict of Interest Statement

The authors declare that the research was conducted in the absence of any commercial or financial relationships that could be construed as a potential conflict of interest.

## References

[B1] Alliot F. Godin I. Pessac B. (1999). Microglia derive from progenitors, originating from the yolk sac, and which proliferate in the brain. *Brain Res. Dev. Brain Res.* 117 145–152 10.1016/s0165-3806(99)00113-3 10567732

[B2] Antony J. M. Paquin A. Nutt S. L. Kaplan D. R. Miller F. D. (2011). Endogenous microglia regulate development of embryonic cortical precursor cells. *J. Neurosci. Res.* 89 286–298 21259316 10.1002/jnr.22533

[B3] Bachstetter A. D. Morganti J. M. Jernberg J. Schlunk A. Mitchell S. H. Brewster K. W. (2011). Fractalkine and CX3CR1 regulate hippocampal neurogenesis in adult and aged rats. *Neurobiol. Aging* 32 2030–2044 20018408 10.1016/j.neurobiolaging.2009.11.022 PMC2889032

[B4] Barclay A. N. Wright G. J. Brooke G. Brown M. H. (2002). CD200 and membrane protein interactions in the control of myeloid cells. *Trends Immunol.* 23 285–290 12072366 10.1016/s1471-4906(02)02223-8

[B5] Bazan J. F. Bacon K. B. Hardiman G. Wang W. Soo K. Rossi D. (1997). A new class of membrane-bound chemokine with a CX3C motif. *Nature* 385 640–644 9024663 10.1038/385640a0

[B6] Beers D. R. Henkel J. S. Xiao Q. Zhao W. Wang J. Yen A. A. (2006). Wild-type microglia extend survival in PU.1 knockout mice with familial amyotrophic lateral sclerosis. *Proc. Natl. Acad. Sci. U.S.A.* 103 16021–16026 17043238 10.1073/pnas.0607423103 PMC1613228

[B7] Biber K. Neumann H. Inoue K Boddeke H. W. G. M. (2007). Neuronal “On” and “Off” signals control microglia. *Trends Neurosci.* 30 596–602 17950926 10.1016/j.tins.2007.08.007

[B8] Blevins G. Fedoroff S. (1995). Microglia in colony-stimulating factor 1-deficient op/op mice. *J. Neurosci. Res.* 40 535–544 7616613 10.1002/jnr.490400412

[B9] Cardona A. E. Pioro E. P. Sasse M. E. Kostenko V. Cardona S. M. Dijkstra I. M. (2006). Control of microglial neurotoxicity by the fractalkine receptor. *Nat. Neurosci.* 9 917–924 16732273 10.1038/nn1715

[B10] Cecchini M. G. Dominguez M. G. Mocci S. Wetterwald A. Felix R. Fleisch H. (1994). Role of colony stimulating factor-1 in the establishment and regulation of tissue macrophages during postnatal development of the mouse. *Development* 120 1357–1372 8050349 10.1242/dev.120.6.1357

[B11] Chen S. -K. Tvrdik P. Peden E. Cho S. Wu S. Spangrude G. (2010). Hematopoietic origin of pathological grooming in Hoxb8 mutant mice. *Cell* 141 775–785 20510925 10.1016/j.cell.2010.03.055 PMC2894573

[B12] Chitu V. Stanley E. R. (2006). Colony-stimulating factor-1 in immunity and inflammation. *Curr. Opin. Immunol.* 18 39–48 16337366 10.1016/j.coi.2005.11.006

[B13] Colonna M. (2003). TREMs in the immune system and beyond. *Nat. Rev. Immunol.* 3 445–453 12776204 10.1038/nri1106

[B14] Cuadros M. A Navascués J. (1998). The origin and differentiation of microglial cells during development. *Prog. Neurobiol.* 56 173–189 9760700 10.1016/s0301-0082(98)00035-5

[B15] Dai X.-M. Ryan G. R. Hapel A. J. Dominguez M. G. Russell R. G. Kapp S. (2002). Targeted disruption of the mouse colony-stimulating factor 1 receptor gene results in osteopetrosis, mononuclear phagocyte deficiency, increased primitive progenitor cell frequencies, and reproductive defects. *Blood* 99 111–120 11756160 10.1182/blood.v99.1.111

[B16] Davalos D. Grutzendler J. Yang G. Kim J. V. Zuo Y. Jung S. (2005). ATP mediates rapid microglial response to local brain injury in vivo. *Nat. Neurosci.* 8 752–758 15895084 10.1038/nn1472

[B17] De Jager P. L. Jia X. Wang J. de Bakker P. I. W. Ottoboni L. Aggarwal N. T. (2009). Meta-analysis of genome scans and replication identify CD6, IRF8 and TNFRSF1A as new multiple sclerosis susceptibility loci. *Nat. Genet.* 41 776–782 19525953 10.1038/ng.401 PMC2757648

[B18] del Rio-Hortega P. (1919). El “tercer elemento” de los centros nerviosus. I. La microglia en estado normal. II. Intervencion de la microglia en los procesos patologicos (Celulas en bastoncito y cuerpos granuloadiposos). III. Naturaleza probable de la microglia. *Bol. R. Soc. Esp. Hist. Nat. Secc. Biol.* 9 68–120

[B20] del Rio-Hortega P. (1932). “Microglia,” in *Cytology and Cellular Pathology of the Nervous System* ed Penfield W. (New York: P. B. Hoeber) 483–534

[B19] Erblich B. Zhu L. Etgen A. M. Dobrenis K. Pollard J. W. (2011). Absence of colony stimulation factor-1 receptor results in loss of microglia, disrupted brain development and olfactory deficits. *PLoS ONE* 6:e26317 10.1371/journal.pone.0026317 PMC3203114 22046273

[B21] Fuhrmann M. Bittner T. Jung C. K. E. Burgold S. Page R. M. Mitteregger G. (2010). Microglial Cx3cr1 knockout prevents neuron loss in a mouse model of Alzheimer’s disease. *Nat. Neurosci.* 13 411–413 20305648 10.1038/nn.2511 PMC4072212

[B22] Ginhoux F. Greter M. Leboeuf M. Nandi S. See P. Gokhan S. (2010). Fate mapping analysis reveals that adult microglia derive from primitive macrophages. *Science* 330 841–845 20966214 10.1126/science.1194637 PMC3719181

[B23] Gómez-Nicola D. Fransen N. L. Suzzi S. Perry V. H. (2013). Regulation of microglial proliferation during chronic neurodegeneration. *J. Neurosci.* 33 2481–2493 23392676 10.1523/JNEUROSCI.4440-12.2013 PMC6619184

[B24] Greer J. M. Capecchi M. R. (2002). Hoxb8 is required for normal grooming behavior in mice. *Neuron* 33 23–34 11779477 10.1016/s0896-6273(01)00564-5

[B25] Greter M. Lelios I. Pelczar P. Hoeffel G. Price J. Leboeuf M. (2012). Stroma-derived interleukin-34 controls the development and maintenance of langerhans cells and the maintenance of microglia. *Immunity* 37 1050–1060 23177320 10.1016/j.immuni.2012.11.001 PMC4291117

[B26] Hambleton S. Salem S. Bustamante J. Bigley V. Boisson-Dupuis S. Azevedo J. (2011). IRF8 mutations and human dendritic-cell immunodeficiency. *N. Engl. J. Med.* 365 127–138 21524210 10.1056/NEJMoa1100066 PMC3136554

[B27] Hanisch U.-K. Kettenmann H. (2007). Microglia: active sensor and versatile effector cells in the normal and pathologic brain. *Nat. Neurosci.* 10 1387–1394 17965659 10.1038/nn1997

[B28] Harrison J. K. Jiang Y. Chen S. Xia Y. Maciejewski D. McNamara R. K. (1998). Role for neuronally derived fractalkine in mediating interactions between neurons and CX3CR1-expressing microglia. *Proc. Natl. Acad. Sci. U.S.A.* 95 10896–10901 9724801 10.1073/pnas.95.18.10896 PMC27992

[B29] Haynes S. E. Hollopeter G. Yang G. Kurpius D. Dailey M. E. Gan W.-B. (2006). The P2Y12 receptor regulates microglial activation by extracellular nucleotides. *Nat. Neurosci.* 9 1512–1519 17115040 10.1038/nn1805

[B30] Hoek R. M. Ruuls S. R. Murphy C. A. Wright G. J. Goddard R. Zurawski S. M. (2000). Down-regulation of the macrophage lineage through interaction with OX2 (CD200). *Science* 290 1768–1771 11099416 10.1126/science.290.5497.1768

[B31] Holtschke T. Löhler J. Kanno Y. Fehr T. Giese N. Rosenbauer F. (1996). Immunodeficiency and chronic myelogenous leukemia-like syndrome in mice with a targeted mutation of the ICSBP gene. *Cell* 87 307–317 8861914 10.1016/s0092-8674(00)81348-3

[B32] Horiuchi M. Wakayama K. Itoh A. Kawai K. Pleasure D. Ozato K. (2012). Interferon regulatory factor 8/interferon consensus sequence binding protein is a critical transcription factor for the physiological phenotype of microglia. *J. Neuroinflammation* 9 227 10.1186/1742-2094-9-227 PMC3546867 23020843

[B33] Hoshiko M. Arnoux I. Avignone E. Yamamoto N. Audinat E. (2012). Deficiency of the microglial receptor CX3CR1 impairs postnatal functional development of thalamocortical synapses in the barrel cortex. *J. Neurosci.* 32 15106–15111 23100431 10.1523/JNEUROSCI.1167-12.2012 PMC6704837

[B34] Hua J. Y. Smith S. J. (2004). Neural activity and the dynamics of central nervous system development. *Nat. Neurosci.* 7 327–332 15048120 10.1038/nn1218

[B35] Hughes P. M. Botham M. S. Frentzel S. Mir A. Perry V. H. (2002). Expression of fractalkine (CX3CL1) and its receptor, CX3CR1, during acute and chronic inflammation in the rodent CNS. *Glia* 37 314–327 11870871

[B36] Inoue K. (2002). Microglial activation by purines and pyrimidines. *Glia* 40 156–163 12379903 10.1002/glia.10150

[B37] Jin H. Li L. Xu J. Zhen F. Zhu L. Liu P. P. (2012). Runx1 regulates embryonic myeloid fate choice in zebrafish through a negative feedback loop inhibiting Pu.1 expression. *Blood* 119 5239–5249 22493295 10.1182/blood-2011-12-398362 PMC3369614

[B38] Jung S. Aliberti J. Graemmel P. Sunshine M. J. Kreutzberg G. W. Sher A. (2000). Analysis of fractalkine receptor CX3CR1 function by targeted deletion and green fluorescent protein reporter gene insertion. *Mol. Cell. Biol.* 20 4106–4114 10805752 10.1128/mcb.20.11.4106-4114.2000 PMC85780

[B39] Kaur C. Hao A. J. Wu C. H. Ling E. A. (2001). Origin of microglia. *Microsc. Res. Tech.* 54 2–9 11526953 10.1002/jemt.1114

[B40] Kierdorf K. Erny D. Goldmann T. Sander V. Schulz C. Perdiguero E. G. (2013). Microglia emerge from erythromyeloid precursors via Pu.1- and Irf8-dependent pathways. *Nat. Neurosci.* 16 273–280 23334579 10.1038/nn.3318

[B41] Kim K.-W. Vallon-Eberhard A. Zigmond E. Farache J. Shezen E. Shakhar G. (2011). In vivo structure/function and expression analysis of the CX3C chemokine fractalkine. *Blood* 118 e156–e167 21951685 10.1182/blood-2011-04-348946 PMC4507037

[B42] Klünemann H. H. Ridha B. H. Magy L. Wherrett J. R. Hemelsoet D. M. Keen R. W. (2005). The genetic causes of basal ganglia calcification, dementia, and bone cysts: DAP12 and TREM2. *Neurology* 64 1502–1507 15883308 10.1212/01.WNL.0000160304.00003.CA

[B43] Lawson L. J. Perry V. H. Dri P. Gordon S. (1990). Heterogeneity in the distribution and morphology of microglia in the normal adult mouse brain. *Neuroscience* 39 151–170 2089275 10.1016/0306-4522(90)90229-w

[B44] Lee S. Varvel N. H. Konerth M. E. Xu G. Cardona A. E. Ransohoff R. M. (2010). CX3CR1 deficiency alters microglial activation and reduces beta-amyloid deposition in two Alzheimer’s disease mouse models. *Am. J. Pathol.* 177 2549–2562 20864679 10.2353/ajpath.2010.100265 PMC2966811

[B45] Lessard C. J. Adrianto I. Ice J. A. Wiley G. B. Kelly J. A. Glenn S. B. (2012). Identification of IRF8, TMEM39A, and IKZF3-ZPBP2 as susceptibility loci for systemic lupus erythematosus in a large-scale multiracial replication study. *Am. J. Hum. Genet.* 90 648–660 22464253 10.1016/j.ajhg.2012.02.023 PMC3322228

[B46] Lin H. Lee E. Hestir K. Leo C. Huang M. Bosch E. (2008). Discovery of a cytokine and its receptor by functional screening of the extracellular proteome. *Science* 320 807–811 18467591 10.1126/science.1154370

[B47] Linnartz B. Neumann H. (2012). Microglial activatory (immunoreceptor tyrosine-based activation motif)- and inhibitory (immunoreceptor tyrosine-based inhibition motif)-signaling receptors for recognition of the neuronal glycocalyx. *Glia* 61 37–46 22615186 10.1002/glia.22359

[B48] Liu Z. Condello C. Schain A. Harb R. Grutzendler J. (2010). CX3CR1 in microglia regulates brain amyloid deposition through selective protofibrillar amyloid-β phagocytosis. *J. Neurosci.* 30 17091–17101 21159979 10.1523/JNEUROSCI.4403-10.2010 PMC3077120

[B49] Masuda T. Tsuda M. Yoshinaga R. Tozaki-Saitoh H. Ozato K. Tamura T. (2012). IRF8 is a critical transcription factor for transforming microglia into a reactive phenotype. *Cell Rep.* 1 334–340 22832225 10.1016/j.celrep.2012.02.014 PMC4158926

[B50] McKercher S. R. Torbett B. E. Anderson K. L. Henkel G. W. Vestal D. J. Baribault H. (1996). Targeted disruption of the PU.1 gene results in multiple hematopoietic abnormalities. *EMBO J.* 15 5647–5658 8896458 PMC452309

[B51] Minten C. Terry R. Deffrasnes C. King N. J. C. Campbell I. L. (2012). IFN regulatory factor 8 is a key constitutive determinant of the morphological and molecular properties of microglia in the CNS. *PLoS ONE * 7:e49851 10.1371/journal.pone.0049851 PMC3498170 23166780

[B52] Mucenski M. L. McLain K. Kier A. B. Swerdlow S. H. Schreiner C. M. Miller T. A. (1991). A functional c-myb gene is required for normal murine fetal hepatic hematopoiesis. *Cell* 65 677–689 1709592 10.1016/0092-8674(91)90099-k

[B53] Neumann H. Takahashi K. (2007). Essential role of the microglial triggering receptor expressed on myeloid cells-2 (TREM2) for central nervous tissue immune homeostasis. *J. Neuroimmunol.* 184 92–99 17239445 10.1016/j.jneuroim.2006.11.032

[B54] Nimmerjahn A. Kirchhoff F. Helmchen F. (2005). Resting microglial cells are highly dynamic surveillants of brain parenchyma in vivo. *Science* 308 1314–1318 15831717 10.1126/science.1110647

[B55] North T. Gu T. L. Stacy T. Wang Q. Howard L. Binder M. (1999). Cbfa2 is required for the formation of intra-aortic hematopoietic clusters. *Development* 126 2563–2575 10226014 10.1242/dev.126.11.2563

[B56] Ohsawa K. Irino Y. Nakamura Y. Akazawa C. Inoue K. Kohsaka S. (2007). Involvement of P2X4 and P2Y12 receptors in ATP-induced microglial chemotaxis. *Glia* 55 604–616 17299767 10.1002/glia.20489

[B57] Okuda T. van Deursen J. Hiebert S. W. Grosveld G. Downing J. R. (1996). AML1, the target of multiple chromosomal translocations in human leukemia, is essential for normal fetal liver hematopoiesis. *Cell* 84 321–330 8565077 10.1016/s0092-8674(00)80986-1

[B58] Paloneva J. Autti T. Raininko R. Partanen J. Salonen O. Puranen M. (2001). CNS manifestations of Nasu–Hakola disease: a frontal dementia with bone cysts. *Neurology* 56 1552–1558 11402114 10.1212/wnl.56.11.1552

[B59] Pan Y. Lloyd C. Zhou H. Dolich S. Deeds J. Gonzalo J.-A. (1997). Neurotactin, a membrane-anchored chemokine upregulated in brain inflammation. *Nature* 387 611–617 9177350 10.1038/42491

[B60] Paolicelli R. C. Bolasco G. Pagani F. Maggi L. Scianni M. Panzanelli P. (2011). Synaptic pruning by microglia is necessary for normal brain development. *Science* 333 1456–1458 21778362 10.1126/science.1202529

[B61] Parakalan R. Jiang B. Nimmi B. Janani M. Jayapal M. Lu J. (2012). Transcriptome analysis of amoeboid and ramified microglia isolated from the corpus callosum of rat brain. *BMC Neurosci.* 13:64 10.1186/1471-2202-13-64 PMC3441342 22697290

[B62] Ponomarev E. D. Veremeyko T. Barteneva N. Krichevsky A. M. Weiner H. L. (2011). MicroRNA-124 promotes microglia quiescence and suppresses EAE by deactivating macrophages via the C/EBP-α-PU.1 pathway. *Nat. Med.* 17 64–70 21131957 10.1038/nm.2266 PMC3044940

[B63] Prinz M. Priller J. (2010). Tickets to the brain: role of CCR2 and CX3CR1 in myeloid cell entry in the CNS. *J. Neuroimmunol.* 224 80–84 20554025 10.1016/j.jneuroim.2010.05.015

[B64] Prinz M. Priller J. Sisodia S. S. Ransohoff R. M. (2011). Heterogeneity of CNS myeloid cells and their roles in neurodegeneration. *Nat. Neurosci.* 14 1227–1235 21952260 10.1038/nn.2923

[B65] Rademakers R. Baker M. Nicholson A. M. Rutherford N. J. Finch N. Soto-Ortolaza A. (2012). Mutations in the colony stimulating factor 1 receptor (CSF1R) gene cause hereditary diffuse leukoencephalopathy with spheroids. *Nat. Genet.* 44 200–205 22197934 10.1038/ng.1027 PMC3267847

[B66] Ransohoff R. M. Perry V. H. (2009). Microglial physio- logy: unique stimuli, specialized responses. *Annu. Rev. Immunol.* 27 119–145 19302036 10.1146/annurev.immunol.021908.132528

[B67] Rogers J. T. Morganti J. M. Bachstetter A. D. Hudson C. E. Peters M. M. Grimmig B. A. (2011). CX3CR1 deficiency leads to impairment of hippocampal cognitive function and synaptic plasticity. *J. Neurosci.* 31 16241–16250 22072675 10.1523/JNEUROSCI.3667-11.2011 PMC3236509

[B68] Saijo K. Glass C. K. (2011). Microglial cell origin and phenotypes in health and disease. *Nat. Rev. Immunol.* 11 775–787 22025055 10.1038/nri3086

[B69] Samokhvalov I. M. Samokhvalova N. I. Nishikawa S. (2007). Cell tracing shows the contribution of the yolk sac to adult haematopoiesis. *Nature* 446 1056–1061 17377529 10.1038/nature05725

[B70] Schulz C. Perdiguero E. G. Chorro L. Szabo-Rogers H. Cagnard N. Kierdorf K. (2012). A lineage of myeloid cells independent of Myb and hematopoietic stem cells. *Science* 336 86–90 22442384 10.1126/science.1219179

[B71] Scott E. W. Simon M. C. Anastasi J. Singh H. (1994). Requirement of transcription factor PU.1 in the development of multiple hemato- poietic lineages. *Science* 265 1573–1577 8079170 10.1126/science.8079170

[B72] Takahashi K. Prinz M. Stagi M. Chechneva O. Neumann H. (2007). TREM2-transduced myeloid precursors mediate nervous tissue debris clearance and facilitate recovery in an animal model of multiple sclerosis. *PLoS Med.* 4:e124 10.1371/journal.pmed.0040124 PMC1851623 17425404

[B73] The International Multiple Sclerosis Genetics Consortium. (2011). The genetic association of variants in CD6, TNFRSF1A and IRF8 to multiple sclerosis: a multicenter case–control study. *PLoS ONE* 6:e18813 10.1371/journal.pone.0018813 PMC3084233 21552549

[B74] Ulmann L. Hatcher J. P. Hughes J. P. Chaumont S. Green P. J. Conquet F. (2008). Up-regulation of P2X4 receptors in spinal microglia after peripheral nerve injury mediates BDNF release and neuropathic pain. *J. Neurosci.* 28 11263–11268 18971468 10.1523/JNEUROSCI.2308-08.2008 PMC6671487

[B75] van Beek E. M. Cochrane F. Barclay A. N van den Berg T. K. (2005). Signal regulatory proteins in the immune system. *J. Immunol.* 175 7781–7787 16339510 10.4049/jimmunol.175.12.7781

[B76] Wang Q. Stacy T. Binder M. Marin-Padilla M. Sharpe A. H. Speck N. A. (1996). Disruption of the Cbfa2 gene causes necrosis and hemorrhaging in the central nervous system and blocks definitive hematopoiesis. *Proc. Natl. Acad. Sci.U.S.A.* 93 3444–3449 8622955 10.1073/pnas.93.8.3444 PMC39628

[B77] Wang Y. Szretter K. J. Vermi W. Gilfillan S. Rossini C. Cella M. (2012). IL-34 is a tissue-restricted ligand of CSF1R required for the development of Langerhans cells and microglia. *Nat. Immunol.* 13 753–760 22729249 10.1038/ni.2360 PMC3941469

[B78] Wei S. Nandi S. Chitu V. Yeung Y.-G. Yu W. Huang M. (2010). Functional overlap but differential expression of CSF-1 and IL-34 in their CSF-1 receptor-mediated regulation of myeloid cells. *J. Leukoc. Biol.* 88 495–505 20504948 10.1189/jlb.1209822 PMC2924605

[B79] Yoshida H. Hayashi S. Kunisada T. Ogawa M. Nishikawa S. Okamura H. (1990). The murine mutation osteopetrosis is in the coding region of the macrophage colony stimulating factor gene. *Nature* 345 442–444 2188141 10.1038/345442a0

[B80] Zusso M. Methot L. Lo R. Greenhalgh A. D. David S. Stifani S. (2012). Regulation of postnatal forebrain amoeboid microglial cell proliferation and development by the transcription factor runx1. *J. Neurosci.* 32 11285–11298 22895712 10.1523/JNEUROSCI.6182-11.2012 PMC6621177

